# Proprietary Medicines

**Published:** 1905-03

**Authors:** 


PROPRIETARY MEDICINES.—Dr. Hadra's paper in this issue should be read and pondered well. It will appeal to every fair-minded and unprejudiced person. It shows up the lack of wisdom in the unjust wholesale and indiscriminating condemnation of every kind of patented or trade mark preparations. I have not seen the committee report, but I infer from Dr. Hadra's paper that it condemns without discrimination all medicines not "put up" according to the physician's prescription. I commend Hadra's courage in thus standing up for his convictions. I endorse them fully. In my September number, it will be remembered, I took about the same view of it. Wholesale pharmacy means an enlightened progress. We have gotten out of the ox-cart days of the "dispensing druggist." He is a back number. The doctor should carry and dispense his own medicines, and legitimate and honorable manufacturers will supply him with articles of uniform strength and purity.

